# Impact of Total Body Surface Area Burn Injuries on Clinical Outcomes and Comorbidities in Elderly Patients Aged Over 65

**DOI:** 10.7759/cureus.76253

**Published:** 2024-12-23

**Authors:** Armein Rahimpour, Nathan Fox, Jamie Anderson, Christina M Arcand, Pranav Balakrishnan, David Denning, Farzad Amiri, Curtis W Harrison, Paul Bown, Rahman Barry

**Affiliations:** 1 General Surgery, Marshall University Joan C. Edwards School of Medicine, Huntington, USA; 2 Plastic and Reconstructive Surgery, Marshall University Joan C. Edwards School of Medicine, Huntington, USA

**Keywords:** appalachia, burn injuries, burn outcomes, elderly patients, resource allocation, tbsa

## Abstract

Introduction: Burn injuries are associated with high mortality and morbidity, especially in the elderly population. Although burns are preventable, they account for the fourth most common cause of trauma worldwide. The majority of the mortality associated with burn victims is also seen in the elderly age group. Most mortality predictor scores focus on age and total body surface area (TBSA) burned. However, data focusing specifically on elderly populations with respect to TBSA, particularly within the Appalachian region, remain limited. This rough terrain is accompanied by multiple challenges and health care disparities with limited burn care access. The population was a significant portion of the elderly who have multiple comorbidities; the majority of the population are economically struggling, living in rural communities, and the state of West Virginia (WV) is considered to have the highest drug use/addiction in the country. It is not shocking that they have the worst health outcomes in the nation.

Aim: This study aims to evaluate the impact of TBSA burn on clinical outcomes and comorbidities in elderly burn patients within Appalachia.

Materials and methods: Cabell Huntington Hospital, the only burn intensive care unit (BICU) in WV, was investigated in this retrospective study. This cohort study analyzed data from 198 patients aged 65 and older admitted to the BICU between January 2017 and January 2023. Data included demographic variables, TBSA burned, comorbidities, and outcomes. Statistical analyses assessed relationships between TBSA and age, gender, length of hospital stay, discharge status, chronic obstructive pulmonary disease (COPD), smoking history, diabetes mellitus (DM), BMI, and inhalation injury. Different statistical analyses were used to analyze the relationship between TBSA and the variables of interest.

Results: Our result section indicated that the majority of the elderly patients with burns were males (65%); however, there was no statistical difference between genders and TBSA (p=0.86). The group with higher TBSA was more likely to have COPD (p<0.0001), use home oxygen (p<0.0001), and have inhalation injury on presentation (p=0.002). Older age was associated with higher TBSA burn (p=0.003), with each one-year increase in age, TBSA burned in our population increased by 0.46% (p=0.002). The group with higher TBSA had higher mortality with a significance (p<0.0001). The annual mortality rate for burn victims above the age of 65 in the Appalachia sole BICU is 14 patients per 100. Our study was not able to find any significance for hospital duration, source of burn, presence of DM, or BMI with TBSA burned.

Conclusion: This study in a unique population base will allow clinicians to understand the elderly burn victim in this underserved area of Appalachia, resource-limited and comorbidity-burdened population. This will allow for targeted interventions to improve outcomes in this vulnerable demographic.

## Introduction

Based on the World Health Organization (WHO), nearly 200 thousand burn victims annually die [1]. For non-fatal burn injuries, the morbidity associated with the injury is significant and at times irreversible [1-2]. Although burns are preventable, they account for the fourth most common cause of trauma worldwide [2]. The majority of the mortality associated with burn victims is also seen in the elderly age group [3]. This susceptibility is mainly related to these groups' deterioration in dexterity, coordination, balance, judgment, and cognition [4]. In the older age population, comorbidities play a major factor as well. Patients with epilepsy/seizures, neuropathic conditions such as diabetes with insensate extremities, polypharmacy, dementia, and neuromotor dysfunction are at higher risk [4-6]. Multiple different mortality prediction tools have been created in an effort to predict burn outcomes, which mainly focus on age, and total body surface area (TBSA) burned [7-8]. The extent of burn injury, quantified as TBSA burned, is a critical determinant of patient outcomes [9]. Larger TBSA burns correlate with prolonged hospital stays, higher complication rates, and increased mortality [10]. However, data focusing specifically on elderly populations with respect to TBSA, particularly within the Appalachian region, remain limited.

We have a unique patient population. The state of West Virginia (WV) is entirely located in the Appalachian mountains. This rough terrain is accompanied by multiple challenges and healthcare disparities with limited access [11]. A significant portion of the elderly in this population has multiple comorbidities including morbid obesity, diabetes mellitus (DM), cardiovascular disease such as coronary artery disease (CAD)/peripheral artery disease (PAD), and cancer [11-14]. The majority of the population is economically struggling, and living in rural communities, and the state of WV is considered to be the highest drug use/addiction in the country [11-13]. It has been shown that the population living in rural regions has decreased physical activity and lower socioeconomic [15]. A significant number of burn victims are from low-middle-income households [1]. More than two-thirds of WV adults are overweight or obese [9,13]. It is not shocking that they have the worst health outcomes in the nation [16]. Unfortunately with all these pre-existing conditions, the limit to burn care is poor and the state of WV only has one burn intensive care unit (BICU) at Cabell Huntington Hospital (CHH) [16]. This BICU has only six beds, underscoring its critical role in burn care across the state. Due to the limited beds available, it is of high importance to understand the effect of TBSA on patient outcomes in an effort to resource allocation and optimization.

This study aims to evaluate the impact of TBSA burn on clinical outcomes and comorbidities in elderly burn patients within Appalachia. By analyzing variables such as age, gender, hospital length of stay, discharge status, presence of chronic obstructive pulmonary disease (COPD), home oxygen use, smoking history, DM, burn source, inhalation injury, and body mass index (BMI), we seek to identify factors that significantly affect prognosis in this vulnerable demographic. Understanding these relationships is essential for developing targeted interventions to improve survival and quality of life among elderly burn patients.

## Materials and methods

The study received approval from the Marshall University Institutional Review Board (IRB no. 2063568-1). Patient records were retrospectively reviewed from a centralized hospital registry. The hospital is a regional referral center and a teaching institution, offering specialized burn care. The analyzed dataset included patients admitted between January 1, 2017, and January 1, 2023. Data were obtained from the hospital's information technology department, which provided comprehensive information on patients presenting with burns during the study period.

Data collection

Patient data included demographic information (age, gender, and BMI), clinical variables (hospital length of stay, discharge status), comorbid conditions (history of COPD, home oxygen use, smoking history, DM), burn-specific details (source of burn, inhalation injury), and TBSA burned. The TBSA was calculated using the Wallace Rule of Nines. Inhalation injury was diagnosed based on the presence of carbonaceous material or soot in the oropharynx with associated difficulty in oxygenation. Most patients had bronchoscopy; however, it was not a criterion for diagnosis. Burn sources were categorized into the following types: thermal contact burns (e.g., metal), thermal explosion burns, thermal fire burns, thermal flash burns, and thermal scald burns. Exclusion criteria included patients misdiagnosed with burns, such as those with Stevens-Johnson syndrome, frostbite, or road rash, as well as cases lacking documented evidence of burns. After exclusion, the final sample consisted of 198 patients aged 65 years and older.

Statistical analysis

Descriptive statistics were used to summarize the sample characteristics. Continuous variables were reported as mean ± standard deviation (SD), and categorical variables were presented as numbers (N) and percentages (%). The relationships between TBSA (dichotomized as above or below the median classified as low vs. high TBSA) and variables were assessed using chi-square tests. ANOVA was used to determine if there were statistically significant differences in TBSA among the burn source categories. A p-value of less than 0.05 was considered statistically significant. All data analysis was conducted using Python for statistical computing.

## Results

Demographic characteristics

The cohort consisted of 198 individuals, with a gender distribution of 129 males (65%) and 69 females (35%). Gender proportions were consistent across all subgroups, with no statistically significant differences observed based on TBSA categories (p=0.86). The mean age of the cohort was 72.6 years (SD: ±6.1). Patients with higher TBSA (above the median of 5%) were slightly older on average (mean: 74.2, SD: ±6.5) compared to those with lower TBSA (mean: 71.3, SD: ±5.7) (p=0.003). This finding highlights an association between advancing age and higher TBSA in burn injuries. A linear regression model was used to quantify the relationship between age and TBSA burned. The model showed that each one-year increase in age is associated with an increase of 0.46% in TBSA burned (β=0.461, p=0.002).

Clinical outcomes and hospital stay

The average total hospital stay for the entire cohort was 11.7 days (SD: ±12.1). Patients with higher TBSA exhibited a longer mean hospital stay (14.9 days, SD: ±13.8) compared to those with lower TBSA (8.4 days, SD: ±9.1); however, this difference was not statistically significant (p=0.54). Discharge status revealed significant differences related to TBSA. The majority of patients (170, 86%) survived, while 28 (14%) succumbed to their injuries. Deceased patients had a substantially higher mean TBSA (mean: 32.8%, SD: ±20.1%) compared to survivors (mean: 6.7%, SD: ±6.2%) (p<0.0001).

Comorbidities

Diabetes Mellitus

DM was present in 67 patients (33%). Among patients with higher TBSA, DM prevalence was 30 (32%) compared to 37 (36%) in those with lower TBSA (p=0.6206).

Chronic Obstructive Pulmonary Disease

COPD was present in 52 patients (26%). Among patients with higher TBSA, COPD prevalence was significantly higher (38%) compared to those with lower TBSA (14%) (p<0.0001).

Home Oxygen Use

A significant association was observed between TBSA and home oxygen use, with 42% of patients in the high TBSA group requiring oxygen therapy, compared to only 15% in the low TBSA group (p<0.0001).

Smoking History

Smoking history was documented in 44% of the cohort. Patients with higher TBSA demonstrated a greater prevalence of smoking history (61%) compared to 28% in the lower TBSA group (p<0.0001).

Body Mass Index

The mean BMI for the cohort was 28.8 (SD: ±9.3). There was no significant difference in BMI between patients with higher TBSA (mean: 28.2, SD: ±8.5) and those with lower TBSA (mean: 29.4, SD: ±10.1) (p=0.33).

Burn-related factors

Inhalation Injury

Inhalation injuries were noted in 35 patients (18%). Patients with higher TBSA had a significantly greater incidence of inhalation injuries (28%) compared to 8% in the lower TBSA group (p=0.002).

Burn Source

Thermal burns from fire accounted for the majority (51%) of injuries. No statistically significant relationship was found between burn source and TBSA.

Tables 1-3 provide a detailed summary of the demographic and clinical characteristics of the study cohort.

**Table 1:** Descriptive statistics and analysis for comorbidities, inhalation injury, and gender n: number of patients; %: percentage; χ^2^: chi-square; TBSA: total body surface area High TBSA and low TBSA represent TBSA that is above or below the median of TBSA for the cohort.

Variables	n (%)	High TBSA (%)	Low TBSA (%)	p-value	Test statistic
DM	67 (33)	32	36	p=0.6206	χ^2^=0.25
COPD	52 (26)	38	14	p<0.0001	χ^2^=45.13
Home oxygen use	63 (32)	42	15	p<0.0001	χ^2^=30.25
Smoking history	87 (44%)	61	28	p<0.0001	χ^2^=42.56
Inhalation injury	35 (18)	28	8	p=0.002	χ^2^=1.57
Gender				p=0.86	χ^2^=1.57
Males	129 (65)	52	48		
Females	69 (35)	38	62		

**Table 2:** Descriptive statistics and analysis for age, total hospital duration, and BMI t: t-test value; SD: standard deviation; BMI: body mass index; TBSA: total body surface area High TBSA and low TBSA represent TBSA that is above or below the median of TBSA for the cohort

Variables	Average (SD)	High TBSA	Low TBSA	p-value	Test statistic
Age (years)	72.6 (±6.1)	74.2 (SD: ±6.5)	71.3 (SD: ±5.7)	p=0.003	t=-3.01
Hospital duration (days)	11.7 (±12.1)	14.9 (SD: ±13.8)	8.4 (SD: ±9.1)	p=0.54	t=-5.69
BMI	28.8 (±9.3)	28.2 (SD: ±8.5)	29.4 (SD: ±10.1)	p=0.33	t=-1.05

**Table 3:** Burn sources n: number; %: percentage

Variables	n (%)
Burn source	
Thermal burns from fire	102 (52)
Thermal burns from flash	46 (23)
Thermal burns from scald	34 (17)
Thermal burns from explosion	11 (5.5)
Thermal burns from contact	5 (2.5)

## Discussion

This retrospective study examined the relationship between TBSA and various clinical factors and pre-existing conditions in a unique patient population in the resource-limited Appalachian BICU.

Our result section indicated that the majority of the elderly patients with burns were males. In our cohort, 65% were males and 35% females. Although there was no statistical difference between genders and TBSA (p=0.86), males are more prone to burn injuries even in older age groups. This has been seen in multiple other research and it holds true even in the Appalachia burn population [17-18]. One study showed 73% and another 62% of the population burned were males [17-18]. Furthermore, males have more severe injuries and undergo more surgical intervention with longer hospital stays [17]**. **Future studies have to analyze the gender disparities in surgical interventions, hospital duration, and mortality in our unique population.

One significant finding of our study is patients with older age have a higher TBSA burned (p=0.003). Furthermore linear regression model showed that for each one-year increase in age, TBSA burned in our population increased by 0.46% (p=0.002). This finding will allow our community to be aware and preventive measures can be created to in turn save the subgroup of patients with a higher chance of being burned. Multiple prediction mortality scores have been created that emphasize TBSA and age [7-8]. The Baux score for example only looks at the sum of age and TBSA to predict mortality [8]. Our study shows that the older the patient is, the higher the TBSA, in turn, the higher the mortality. This hypothesis was confirmed by looking at the discharge status between low and high TBSA, where the higher TBSA group had higher mortality with a significance (p<0.0001).

The mortality rate for patients above the age of 65 in our population was 28 patients in total; on average of 4.5 patients a year with a mortality rate of 0.136. The annual mortality rate for burn victims above the age of 65 in the Appalachia sole BICU is 14 patients per 100. This mortality rate is really low compared to other studies. One study showed a mortality rate of 56 patients per 100 for a population above the age of 65 [19]. The study confirmed that the older patient group is at higher risk of mortality with high significance (p<0.0001) [19]. Another study had a more similar mortality rate to our population, 11 patients per 100 [20]. This variation in morality between different studies has to do with the patient population. However, overall national mortality based on the American Burn Association for patients above 65 in the United States is above 25% [21]. This mortality increases with age even within this subgroup [21]. This has to be explained by the poor physiological reserve for this group of population and other cardiovascular and pulmonary comorbidities.

Our study was not able to find any significance for hospital duration, presence of DM or BMI with TBSA burned. More than 25% of our population have COPD, which is more than double the national average [22]. The presence of COPD or home oxygen use in our population was associated with higher TBSA burned (p<0.0001). This finding can be explained by patients in this subgroup lacking the typical reserve and having difficulty ambulating. Oxygen tubing and tank supply also act as barriers and constrictions. Furthermore, home oxygen can act as a source of ignition and increase flammability, in turn increasing TBSA burned. These findings were further supportive with higher TBSA seen in a group of patients with inhalational injury (p=0.002). In our study, 18% had an inhalational injury. Although data is limited on elderly patients with inhalational injury, one study showed 35% inhalational injury [19]. The difference can be explained by the classification and diagnosis of inhalational injury in the elderly population between studies.

Different studies have shown that the increase in TBSA and hospital duration increases as well [23-25]. Although patients with higher TBSA exhibited a longer mean hospital stay compared to those with lower TBSA; this difference was not statistically significant. This finding may be attributed to the unique characteristics of our patient population, where the presence of multiple comorbidities contributes to prolonged hospital stays, irrespective of the burn severity. The national prevalence of diabetes for adults is 11.3% [26], COPD is 6.5% [27], and on average 11% of adults smoke [28]. In our study population, 33% of patients have diabetes, 26% have COPD, 44% are smokers, and the average BMI falls in the overweight category. These factors likely contribute to the prolonged hospital stays observed, as such comorbidities can independently impact recovery and lengthen hospitalization, regardless of burn severity. Patients above the age of 65 are disproportionately at risk for mortality. American Burn Association shows this rate to be 12% for burn victims in the United States [28]. Our patient mortality rate, despite having higher comorbidities and limited resources, is comparable to the national average of 14%.

This study has high significance as it is investigating a unique population in Appalachia at the sole BICU in the state of WV. These findings will allow clinicians to understand the elderly burn victims in Appalachia and for further education and outreach. With limited healthcare resources and specialized facilities in WV, strategic resource allocation is vital to ensure optimal patient outcomes. Educational programs for community and healthcare providers can be beneficial.

Although this novel study is unique and fills the gap in understanding the Appalachian burn victim, it is limited in the sense of being retrospective and a single institution. The retrospective design of this study allows for biases and limits generalizability. This population is really unique. Future studies should be performed in a multicenter prospective design to diversify the population and allow for improved generalization.

## Conclusions

Higher TBSA burned is a significant predictor of adverse outcomes in elderly burn patients. The unique challenges of burn care in Appalachia, including resource limitations and a comorbidity-burdened patient population, highlight the need for targeted interventions to improve outcomes in this vulnerable demographic.
